# Asymptotic aspect of derivations in Banach algebras

**DOI:** 10.1186/s13660-017-1308-0

**Published:** 2017-02-06

**Authors:** Jaiok Roh, Ick-Soon Chang

**Affiliations:** 1 Department of Mathematical Finance and Information Statistics, Hallym University, 1 Hallymdaehakggil, Chuncheon, 24252 Korea; 2 Department of Mathematics, Chungnam National University, 99 Daehangno, Yuseong-gu, Daejeon, 34134 Korea

**Keywords:** 16N20, 16N60, 39B72, 39B82, 46H40, 47H99, derivation, inequality, Banach algebra, stability, radical range

## Abstract

We prove that every approximate linear left derivation on a semisimple Banach algebra is continuous. Also, we consider linear derivations on Banach algebras and we first study the conditions for a linear derivation on a Banach algebra. Then we examine the functional inequalities related to a linear derivation and their stability. We finally take central linear derivations with radical ranges on semiprime Banach algebras and a continuous linear generalized left derivation on a semisimple Banach algebra.

## Introduction and preliminaries

Let $\mathcal {A}$ be an algebra. A linear mapping $\delta: \mathcal {A}\to \mathcal {A}$ is called a left derivation (resp., derivation) if $\delta(xy)=x\delta(y)+y\delta(x)$ (resp., $\delta(xy)=x\delta(y)+\delta(x)y$) is fulfilled for all $x,y \in \mathcal {A}$. A linear mapping $\delta: \mathcal {A}\to \mathcal {A}$ is said to be a left Jordan derivation if $\delta(x^{2})=2x\delta(x)$ holds for all $x \in \mathcal {A}$. A linear mapping $\delta_{1}:\mathcal {A}\to \mathcal {A}$ is called a generalized left derivation if there exists a linear left derivation $\delta_{0}:\mathcal {A}\to \mathcal {A}$ such that $\delta_{1}(xy)=x\delta_{1}(y)+y\delta_{0}(x)$ for all $x,y \in \mathcal {A}$. A linear mapping $\delta_{1}:\mathcal {A}\to \mathcal {A}$ is said to be a generalized left Jordan derivation if there exists a linear left Jordan derivation $\delta_{0}:\mathcal {A}\to \mathcal {A}$ such that $\delta_{1}(x^{2})=x\delta _{1}(x)+x\delta_{0}(x)$ for all $x \in \mathcal {A}$.

Singer and Wermer [1] obtained a fundamental result which started the investigation of the ranges of linear derivations on Banach algebras. The result, which is called the Singer-Wermer theorem, states that every continuous linear derivation on a commutative Banach algebra maps into the radical. In the same paper, they made a very insightful conjecture: that the assumption of continuity is unnecessary. Thomas [2] proved this conjecture. Hence linear derivations on Banach algebras (if everywhere defined) genuinely belong to the noncommutative setting.

On the other hand, the study of stability problems had been formulated by Ulam [3]. Hyers [4] had answered affirmatively the question of Ulam for Banach spaces. Hyers’ theorem was generalized by Aoki [5] for additive mappings and by Rassias [6] for linear mappings by considering an unbounded difference. In particular, the stability result concerning derivations between operator algebras was first obtained by Šemrl [7]. Badora gave a generalization of the Bourgin result and he also dealt with the stability and the superstability of Bourgin-type for derivations; see [8–10] and the references therein. Recently, the stability problems for derivations are considered by some authors in [11–13].

In this work, we first take into account the functional inequality which expands the functional inequality in [14]. It is well known that every ring left derivation (resp., ring left Jordan derivation) on a semiprime ring maps into its center; see [15, 16]. Considering the base of the previous result, we show that every approximate ring left derivation on a semiprime normed algebra maps into its center and then, by using this fact, we prove that every approximate linear left derivation on a semisimple Banach algebra is continuous. We also establish the functional inequalities related to a linear derivation and their stability. In particular, mappings satisfying such functional inequalities on a semiprime Banach algebra are linear derivations which map into the intersection of the center and the radical. We finally investigate a linear generalized left Jordan derivation on a semisimple Banach algebra with application.

## Approximate left derivations

We first demonstrate the following proposition quoted in this work.

### Proposition 2.1

[15], Proposition 1.6

*Let* $\mathcal {R}$ *be a ring*, $\mathcal {X}$ *be a left* $\mathcal {R}$-*module*, *and* $\delta : \mathcal {R}\to \mathcal {X}$ *be a left derivation*.

(i) *Suppose that* $a \mathcal {R}x =0$ *with* $a \in \mathcal {R}, x \in \mathcal {X}$ *implies* $a=0$ *or* $x=0$. *If* $\delta \ne0$, *then* $\mathcal {R}$ *is commutative*.
(ii) *Suppose that* $\mathcal {X}=\mathcal {R}$ *is a semiprime ring*. *Then* *δ* *is a derivation which maps* $\mathcal {R}$ *into its center*.

Let $\mathcal {A}$ be a normed algebra. An additive mapping $\delta :\mathcal {A}\to \mathcal {A}$ is said to be an approximate ring derivation (resp., approximate ring left derivation) if for some $\varepsilon\ge0$,

$$\bigl\Vert \delta (xy)-x\delta (y)-\delta (x)y\bigr\Vert \le\varepsilon \quad\bigl(\mbox{resp.,} \bigl\Vert \delta (xy)-x\delta(y)-y\delta(x)\bigr\Vert \leq\varepsilon\bigr) $$

for all $x,y \in \mathcal {A}$. In addition, if $\delta (\lambda x)=\lambda \delta (x)$ for all $x \in \mathcal {A}$ and $\lambda \in\mathbb{C}$, then *δ* is called an approximate linear derivation (resp., approximate linear left derivation).

From now on, we suppose that $\mathbb{T}_{\varepsilon} :=\{ e^{i \theta}: 0 \leq\theta\leq \varepsilon\}$. The commutator $xy-yx$ will be denoted by $[x,y]$. We start our investigations for approximate ring left derivations with some results.

### Theorem 2.2

*Let* $\mathcal {A}$ *be a semiprime normed algebra*. *Assume that* $l \ge3$ *is a fixed integer and* $s_{1},s_{2}, \ldots, s_{l}$ *are fixed positive real numbers*, *where* $s_{j}> 1$ ($j=1,2$) *and* $s_{3}=1$. *Suppose that* $\delta: \mathcal {A}\to \mathcal {A}$ *is a mapping such that*

2.1 $$\begin{aligned} \Biggl\| \sum_{j=1}^{l}s_{j} \delta(x_{j}) \Biggr\| \leq \Biggl\| \delta \Biggl(\sum _{j=1}^{l}s_{j}x_{j} \Biggr) \Biggr\| \end{aligned}$$

*for all* $x_{1}, x_{2}, \ldots, x_{l}\in \mathcal {A}$ *and for some* $\varepsilon\ge0$,

2.2 $$\begin{aligned} \bigl\Vert \delta(xy)-x\delta(y)-y\delta(x)\bigr\Vert \leq \varepsilon \end{aligned}$$

*for all* $x,y \in \mathcal {A}$. *Then* *δ* *is an approximate ring derivation which maps* $\mathcal {A}$ *into its center* $Z(\mathcal {A})$.

### Proof

By letting $x_{1}=x_{2}=\cdots=x_{l}=0$ in (2.1), we get $\delta(0)=0$. And we put $x_{4}=x_{5}=\cdots=x_{l}=0$ in (2.1) and then set $x_{1}=x, x_{2}=y,x_{3}=z,s_{1}=s,s_{2}=t$ to obtain

2.3 $$\begin{aligned} \bigl\Vert s\delta(x)+t\delta(y)+\delta(z)\bigr\Vert \le\bigl\Vert \delta (sx+ty+z)\bigr\Vert \quad\mbox{for all } x,y,z \in \mathcal {A}. \end{aligned}$$

It follows by the result of [14] that *δ* is additive. In particular, in view of (2.2), we find that *δ* is an approximate ring left derivation.

By virtue of (2.2), we see that

2.4 $$\begin{aligned} \bigl\Vert \delta(yx)-y\delta(x)-x\delta(y)\bigr\Vert \leq \varepsilon \end{aligned}$$

for all $x,y \in \mathcal {A}$. Combining (2.2) and (2.4), we get

2.5 $$\begin{aligned} \bigl\Vert \delta(xy)-\delta(yx)\bigr\Vert \leq\bigl\Vert \delta (xy)-x\delta(y)-y\delta(x)\bigr\Vert +\bigl\Vert \delta(yx)-y \delta(x)-x\delta(y)\bigr\Vert \leq2\varepsilon \end{aligned}$$

for all $x,y \in \mathcal {A}$. It follows from (2.2) and (2.5) that

2.6 $$\begin{aligned} \bigl\Vert [x,y]\delta(x)\bigr\Vert \leq{}&\bigl\Vert \delta(x\cdot yx)-x\delta(yx)-yx\delta (x)\bigr\Vert \\ &{} +\bigl\Vert \delta(xy \cdot x)-xy\delta(x)-x\delta(xy)\bigr\Vert +\| x\| \bigl\| \delta (xy)-\delta(yx)\bigr\| \\ \leq{}&2\varepsilon\bigl(\Vert x\Vert + 1\bigr) \end{aligned}$$

for all $x,y \in \mathcal {A}$. Replacing *x* by *nx* in (2.6) and then dividing on both sides by $n^{2}$, we have

$$\begin{aligned} \bigl\Vert [x,y]\delta(x)\bigr\Vert \leq2 \biggl(\frac{\| x\|}{n} + \frac {1}{n^{2}} \biggr) \varepsilon \end{aligned}$$

for all $x,y \in \mathcal {A}$ and all positive integer *n*. Taking the limit as $n \to\infty$ in the above relation, we see that

2.7 $$\begin{aligned}{} [x,y]\delta(x)=0 \quad\mbox{for all } x,y \in \mathcal {A}. \end{aligned}$$

Just proceeding as in the proof of Proposition 2.1, we get $[\delta(w),x]=0$ for all $x,w \in \mathcal {A}$. That is, $\delta(w)$ belong to its center $Z(\mathcal {A})$. So *δ* is an approximate ring derivation. Therefore we arrive at the desired conclusion. □

### Theorem 2.3

*Let* $\mathcal {A}$ *be a noncommutative prime normed algebra*. *Assume that* $l \ge3$ *is a fixed integer and* $s_{1},s_{2}, \ldots, s_{l}$ *are fixed positive real numbers*, *where* $s_{j}> 1$ ($j=1,2$) *and* $s_{3}=1$. *Suppose that* $\delta: \mathcal {A}\to \mathcal {A}$ *is a mapping subject to the conditions* (2.1) *and* (2.2). *Then* *δ* *is identically zero*.

### Proof

Employing the same argument as the proof Theorem 2.2, we feel that *δ* satisfies equation (2.7). Since $\mathcal {A}$ is noncommutative, choose a *z* that does not belong to the center of $\mathcal {A}$. Using the same method in the proof of Proposition 2.1, we see that $\delta=0$, which completes the proof. □

### Theorem 2.4

*Let* $\mathcal {A}$ *be a semisimple Banach algebra*. *Assume that* $l \ge3$ *is a fixed integer and* $s_{1},s_{2}, \ldots, s_{l}$ *are fixed positive real numbers*, *where* $s_{1}=\lambda s$ ($s>1$), $s_{2}> 1$ *and* $s_{3}=1$. *Suppose that* $\delta: \mathcal {A}\to \mathcal {A}$ *is a mapping subject to*

2.8 $$\begin{aligned} \Biggl\Vert \sum_{j=1}^{l}s_{j} \delta(x_{j})\Biggr\Vert \leq\Biggl\Vert \delta \Biggl(\sum _{j=1}^{l}s_{j}x_{j} \Biggr)\Biggr\Vert \end{aligned}$$

*for all* $x_{1}, x_{2}, \ldots, x_{l}\in \mathcal {A}$ *and all* $\lambda\in \mathbb {T}_{\varepsilon}$, *where* $x_{3}= \lambda z$ ($z \in \mathcal {A}$) *and the inequality* (2.2). *Then* *δ* *is a continuous*.

### Proof

As we did in the proof of Theorem 2.2, we get $\delta(0)=0$. We take $x_{4}=x_{5}=\cdots=x_{l}=0$ in (2.8) and then put $x_{1}=x, x_{2}=y,s_{2}=t$ to have

2.9 $$\begin{aligned} \bigl\Vert \lambda s\delta(x)+t\delta(y)+\delta(\lambda z)\bigr\Vert \le \bigl\Vert \delta (\lambda sx+ty+\lambda z)\bigr\Vert \end{aligned}$$

for all $x,y,z \in \mathcal {A}$ and all $\lambda\in\mathbb{T}_{\varepsilon}$. Now we consider $\lambda=1$ in (2.9) and so *δ* satisfies the inequality (2.3). Hence we find that *δ* is additive [14].

Next, setting $x=\frac{x}{s}, y=0$ and $z=-x$ in (2.3), we obtain $s\delta(\frac{x}{s})=\delta(x)$. Letting $x=\frac{x}{s}, y=0$, and $z=-x$ in (2.9), we get $\delta(\lambda x)=\lambda\delta(x)$ for all $x\in \mathcal {A}$ and all $\lambda\in\mathbb{T}_{\varepsilon}$ and so we see that *δ* is linear [17].

Since semisimple algebras are semiprime [18], Theorem 2.2 guarantees that *δ* is an approximate linear derivation. Therefore *δ* is continuous [14]. The proof is complete. □

## Inequalities related to a linear derivation

In this section, we write a unit element of algebra $\mathcal {A}$ by *e*.

### Theorem 3.1

*Let* $\mathcal {A}$ *be a semiprime unital Banach algebra*. *Suppose that* $\delta: \mathcal {A}\to \mathcal {A}$ *is a mapping subject to the inequality* (2.8) *and for some* $\varepsilon\ge0$,

3.1 $$\begin{aligned} \bigl\Vert \delta\bigl(x^{2}\bigr)-2x\delta(x)\bigr\Vert \leq\varepsilon \end{aligned}$$

*for all* $x \in \mathcal {A}$. *Then* *δ* *is a linear derivation which maps* $\mathcal {A}$ *into the intersection of its center* $Z(\mathcal {A})$ *and its radical* $\operatorname{rad}(\mathcal {A})$.

### Proof

Employing the same way in the proof Theorem 2.4, we find that *δ* is linear. By linearization of (3.1) and additivity of *δ*, we get

3.2 $$\begin{aligned} \bigl\Vert \delta\bigl(x^{2}\bigr)+\delta(xy)+ \delta(yx)+\delta\bigl(y^{2}\bigr)-2x\delta (x)-2x\delta (y)-2y \delta(x)-2y\delta(y)\bigr\Vert \leq\varepsilon \end{aligned}$$

for all $x,y \in \mathcal {A}$. Substituting −*x* for *x* in (3.2), we have

3.3 $$\begin{aligned} \bigl\Vert \delta\bigl(x^{2}\bigr)-\delta(xy)- \delta(yx)+\delta\bigl(y^{2}\bigr)-2x\delta (x)+2x\delta (y)+2y \delta(x)-2y\delta(y)\bigr\Vert \leq\varepsilon \end{aligned}$$

for all $x,y \in \mathcal {A}$. Equations (3.2) and (3.3) yield

$$\begin{aligned} &\bigl\Vert 2\delta(xy)+2\delta(yx)-4x\delta(y)-4y\delta(x)\bigr\Vert \\ &\quad\leq\bigl\Vert \delta\bigl(x^{2}\bigr)+\delta(xy)+\delta(yx)+ \delta \bigl(y^{2}\bigr)-2x\delta (x)-2x\delta(y)-2y\delta(x)-2y \delta(y)\bigr\Vert \\ &\qquad{} +\bigl\Vert \delta\bigl(x^{2}\bigr)-\delta(xy)-\delta(yx)+ \delta \bigl(y^{2}\bigr)-2x\delta (x)+2x\delta(y)+2y\delta(x)-2y \delta(y)\bigr\Vert \leq2\varepsilon \end{aligned}$$

for all $x,y \in \mathcal {A}$. We have therefore

3.4 $$\begin{aligned} \bigl\Vert \delta(xy+yx)-2x\delta(y)-2y\delta(x)\bigr\Vert \leq \varepsilon \quad\mbox{for all } x,y \in \mathcal {A}. \end{aligned}$$

Putting $xy+yx$ for *y* in (3.4), we obtain

3.5 $$\begin{aligned} \bigl\Vert \delta\bigl(x(xy+yx)+(xy+yx)x\bigr)-2x \delta(xy+yx)-2(xy+yx)\delta (x)\bigr\Vert \leq \varepsilon \end{aligned}$$

for all $x,y \in \mathcal {A}$. On the other hand, we have from (3.4) and the equation

$$x(xy+yx)+(xy+yx)x=x^{2}y+yx^{2}+2xyx $$

the result

3.6 $$\begin{aligned} &\bigl\Vert \delta\bigl(x(xy+yx)+(xy+yx)x\bigr)-2x^{2} \delta(y)-2y\delta \bigl(x^{2}\bigr)-2\delta (xyx)\bigr\Vert \\ &\quad= \bigl\Vert \delta\bigl(x^{2}y+yx^{2} \bigr)-2x^{2}\delta(y)-2y\delta \bigl(x^{2}\bigr)\bigr\Vert \leq \varepsilon \end{aligned}$$

for all $x,y \in \mathcal {A}$. By comparing (3.5) and (3.6), we arrive at

3.7 $$\begin{aligned} &\bigl\Vert 2x\delta(xy+yx)+2(xy+yx)\delta(x)-2x^{2} \delta(y)-2y\delta \bigl(x^{2}\bigr)-2\delta(xyx)\bigr\Vert \\ &\quad\leq\bigl\Vert \delta\bigl(x(xy+yx)+(xy+yx)x\bigr)-2x^{2} \delta (y)-2y\delta \bigl(x^{2}\bigr)-2\delta(xyx)\bigr\Vert \\ &\qquad{} +\bigl\Vert \delta\bigl(x(xy+yx)+(xy+yx)x\bigr)-2x\delta (xy+yx)-2(xy+yx)\delta (x)\bigr\Vert \leq2\varepsilon \end{aligned}$$

for all $x,y \in \mathcal {A}$. Applying equation (3.7) with (3.1) and (3.4), we have

3.8 $$\begin{aligned} &\bigl\Vert 2x^{2}\delta(y)+6xy\delta(x)-2yx \delta(x)-2\delta (xyx)\bigr\Vert \\ &\quad\leq2\|x\|\bigl\Vert \delta(xy+yx)-2x\delta(y)-2y\delta (x)\bigr\Vert +2\|y\|\bigl\Vert \delta \bigl(x^{2}\bigr)-2x\delta(x)\bigr\Vert \\ &\qquad{} +\bigl\Vert 2x\delta(xy+yx) +2(xy+yx)\delta(x)-2x^{2} \delta(y)-2y\delta\bigl(x^{2}\bigr)-2\delta (xyz)\bigr\Vert \\ &\quad\leq2\bigl(\Vert x\Vert +\|y\|+1\bigr)\varepsilon \end{aligned}$$

for all $x,y \in \mathcal {A}$. Letting $x=nx, y=ny$ in (3.8) and then dividing the resulting inequality by $n^{3}$, we get

3.9 $$\begin{aligned} \bigl\Vert 2x^{2}\delta(y)+6xy\delta(x)-2yx\delta(x)-2 \delta (xyx)\bigr\Vert \leq 2 \biggl(\frac{\|x\|}{n^{2}}+\frac{\|y\|}{n^{2}}+ \frac{1}{n^{3}} \biggr)\varepsilon \end{aligned}$$

for all $x,y \in \mathcal {A}$ and all positive integers *n*. Taking the limit $n \to\infty$ of (3.9), it is reduced to the equation

3.10 $$\begin{aligned} \delta(xyx)=x^{2}\delta(y)+3xy\delta(x)-yx\delta(x) \quad\mbox{for all } x,y \in \mathcal {A}. \end{aligned}$$

Putting $x=y=z=e$ in (3.10), we get $\delta(e)=0$. Again, considering $y=e$ in (3.10), we easily prove that

$$\delta\bigl(x^{2}\bigr)=2x\delta(x) \quad\mbox{for all } x \in \mathcal {A}. $$

This means that *δ* is a linear left Jordan derivation.

On the other hand, from Vukman’s result [16], we see that *δ* is a linear derivation with $\delta(\mathcal {A}) \subseteq Z(\mathcal {A})$. Since $Z(\mathcal {A})$ is a commutative Banach algebra, the Singer-Wermer theorem tells us that $\delta|_{Z(\mathcal {A})}$ maps $Z(\mathcal {A})$ into $\operatorname{rad}(Z(\mathcal {A}))=Z(\mathcal {A}) \cap \operatorname{rad}(\mathcal {A})$ and thus $\delta^{2}(\mathcal {A}) \subseteq \operatorname{rad}(\mathcal {A})$. Using the semiprimeness of $\operatorname{rad}(\mathcal {A})$ as well as the identity

$$2\delta(x)y\delta(x)= \delta^{2}(xyx)-x\delta^{2}(yx)-\delta ^{2}(xy)x+x\delta^{2}(y)x \quad(x,y \in \mathcal {A}), $$

we have $\delta(\mathcal {A}) \subseteq \operatorname{rad}(\mathcal {A})$. Therefore $\delta(\mathcal {A}) \subseteq Z(\mathcal {A}) \cap \operatorname{rad}(\mathcal {A})$, which concludes the proof. □

As consequences of Theorem 3.1, we get the following.

### Corollary 3.2

*Let* $\mathcal {A}$ *be a unital semisimple Banach algebra*. *Assume that a mapping* $\delta : \mathcal {A}\to \mathcal {A}$ *satisfies the assumptions of Theorem* 3.1. *Then* *δ* *is identically zero*.

Now we consider the result which is needed in the following theorems.

### Lemma 3.3

*Let* $\mathcal {A}$ *be a Banach algebra*. *Suppose that* $\mathcal{L}:\mathcal {A}\times \mathcal {A}\to \mathcal {A}$ *is a bilinear mapping and that* *ξ* *and* *η* *are mappings satisfying* $\mathcal{L}(x,y)= x\xi(y)+ y\eta(x)$ *for all* $x,y \in \mathcal {A}$. *If* $\mathcal {A}$ *is semiprime or unital*, *then* *ξ* *and* *η* *are linear mappings*.

### Proof

Note that, for all $x,y \in \mathcal {A}$ and all $\lambda \in \mathbb{C}$,

$$\begin{aligned} x\xi(\lambda y)+ \lambda y\eta(x)=\mathcal{L}(x, \lambda y)=\lambda \mathcal{L}(x,y)=\lambda \bigl( x \xi(y)+ y\eta(x)\bigr). \end{aligned}$$

Hence we see that, for all $x,y \in \mathcal {A}$,

3.11 $$\begin{aligned} x\bigl(\xi(\lambda y)-\lambda \xi(y)\bigr)=0. \end{aligned}$$

If $\mathcal {A}$ is unital, then we see that $\xi(\lambda y)=\lambda \eta(y)$ by letting $x=e$ in (3.11).

If $\mathcal {A}$ is nonunital, then $\xi(\lambda y)-\lambda \xi(y)$ lies in the right annihilator $\operatorname{ran}(\mathcal {A})$ of $\mathcal {A}$. If $\mathcal {A}$ is semiprime, then $\operatorname{ran}(\mathcal {A})=0$, so that $\xi(\lambda y)=\lambda \xi(y)$ for all $y \in \mathcal {A}$ and all $\lambda \in\mathbb{C}$.

Observe that, for all $x,y,z \in \mathcal {A}$,

$$\begin{aligned} x\xi(y+z)+(y+z)\eta(x)&=\mathcal{L}(x, y+z)=\mathcal {L}(x,y)+\mathcal {L}(x,z) \\ &= x\xi(y)+ y\eta(x)+x\xi(z)+ z\eta(x). \end{aligned}$$

Hence $x(\xi(y+z)-\xi(y)-\xi(z))=0$ for all $y,z \in \mathcal {A}$. As above, we get $\xi(x+z)=\xi(x)+\xi(z)$ for all $x,z \in \mathcal {A}$, so that *ξ* is linear.

Similarly, one can prove that *η* is linear. □

### Theorem 3.4

*Let* $\mathcal {A}$ *be a semiprime Banach algebra*. *Assume that* $l \ge3$ *is a fixed integer and* $s_{1},s_{2}, \ldots, s_{l}$ *are fixed positive real numbers*, *where* $s_{1}=\lambda s$ ($s>1$), $s_{2}> 1$ *and* $s_{3}=1$. *Suppose that* $\delta: \mathcal {A}\to \mathcal {A}$ *is a mapping with* $\delta(0)=0$ *such that*, *for some* $\varepsilon\ge0$,

3.12 $$\begin{aligned} \Biggl\Vert \sum_{j=1}^{l}s_{j} \delta(x_{j})\Biggr\Vert \leq\Biggl\Vert \delta \Biggl(\sum _{j=1}^{l}s_{j}x_{j} \Biggr)\Biggr\Vert +\varepsilon \end{aligned}$$

*for all* $x_{1}, x_{2}, \ldots, x_{l} \in \mathcal {A}$ *and all* $\lambda\in\mathbb{T}_{\varepsilon}$, *where* $x_{3}= \lambda z$ ($z \in \mathcal {A}$) *and*

3.13 $$\begin{aligned} \bigl\Vert \delta(xy+yx)-2x\delta(y)-2y\delta(x)\bigr\Vert \leq\theta \end{aligned}$$

*for some* $\theta\ge0$ *and all* $x,y \in \mathcal {A}$. *Then* *δ* *is a linear derivation which maps* $\mathcal {A}$ *into the intersection of its center* $Z(\mathcal {A})$ *and its radical* $\operatorname{rad}(\mathcal {A})$.

### Proof

We let $x_{4}=x_{5}=\cdots=x_{l}=0$ in (3.12) and then put $x_{1}=x, x_{2}=y, s_{2}=t$ to have

3.14 $$\begin{aligned} \bigl\Vert \lambda s\delta(x)+t\delta(y)+\delta(\lambda z)\bigr\Vert \le \bigl\Vert \delta (\lambda sx+ty+\lambda z)\bigr\Vert +\varepsilon \end{aligned}$$

for all $x,y,z \in \mathcal {A}$ and all $\lambda\in\mathbb{T}_{\varepsilon}$. Now we consider $\lambda=1$ in (3.14). It follows from the result in [14] that there exists a unique additive mapping $\mathcal{D}: \mathcal {A}\to \mathcal {A}$ defined by

3.15 $$\begin{aligned} \mathcal{D}(x):=\lim_{n\to\infty}\frac{\delta(s^{n}x)}{s^{n}} \quad\mbox{for all } x \in \mathcal {A}. \end{aligned}$$

Moreover, $s\mathcal{D}(\frac{x}{s})=\mathcal{D}(x)$ holds for all $x \in \mathcal {A}$.

Letting $x=\frac{x}{s}, y=0$, and $z=-x$ in (3.14), we find that

$$\begin{aligned} \biggl\Vert \lambda s\delta \biggl(\frac{x}{s} \biggr)-\delta(\lambda x) \biggr\Vert \le \varepsilon \end{aligned}$$

for all $x\in \mathcal {A}$ and all $\lambda\in\mathbb{T}_{\varepsilon}$. This implies that

$$\begin{aligned} \lim_{n \to\infty}\frac{1}{s^{n}}\biggl\Vert \lambda s\delta \biggl(\frac {s^{n}x}{s} \biggr)-\delta\bigl(\lambda s^{n}x\bigr)\biggr\Vert \le\lim_{n \to \infty} \frac{\varepsilon}{s^{n}}=0. \end{aligned}$$

Thus $\lambda s\mathcal{D}(\frac{x}{s})=\mathcal{D}(\lambda x)$, so that $\mathcal{D}(\lambda x)=\lambda\mathcal{D}(x)$ for all $x\in \mathcal {A}$ and all $\lambda\in\mathbb{T}_{\varepsilon}$. Thus we see that $\mathcal{D}$ is linear [17].

By (3.13), we see that

$$\begin{aligned} \lim_{n \to\infty}\biggl\Vert \frac{\delta(s^{n}(xy+yx))}{s^{n}}- 2x \delta (y)-2 y \frac{\delta(s^{n}x)}{s^{n}}\biggr\Vert \le\lim_{n \to\infty }\frac {\theta}{s^{n}} =0. \end{aligned}$$

Hence we arrive at

$$\mathcal{D}(xy+yx)=2x\delta(y)+2y\mathcal{D}(x) \quad\mbox{for all } x,y \in \mathcal {A}. $$

It follows from Lemma 3.3 that *δ* is linear. Then we have by (3.15) that $\mathcal {D}=\delta$. Therefore

$$\delta\bigl(x^{2}\bigr)=2x\delta(x) \quad\mbox{for all } x \in \mathcal {A}. $$

That is, *δ* is a linear left Jordan derivation.

The remainder of the proof can be carried out similarly to the corresponding part of Theorem 3.1. □

### Theorem 3.5

*Let* $\mathcal {A}$ *be a unital Banach algebra*. *Assume that* $l \ge3$ *is a fixed integer and* $s_{1},s_{2}, \ldots, s_{l}$ *are fixed positive real numbers*, *where* $s_{1}=\lambda s$ ($s>1$), $s_{2}> 1$ *and* $s_{3}=1$. *Suppose that* $\delta: \mathcal {A}\to \mathcal {A}$ *is a mapping with* $\delta(0)=0$ *such that*, *for some* $\varepsilon\ge0$,

3.16 $$\begin{aligned} \Biggl\Vert \sum_{j=1}^{l}s_{j} \delta(x_{j})\Biggr\Vert \leq\Biggl\Vert \delta \Biggl(\sum _{j=1}^{l}s_{j}x_{j} \Biggr)\Biggr\Vert +\varepsilon \end{aligned}$$

*for all* $x_{1}, x_{2}, \ldots, x_{3} \in \mathcal {A}$ *and all* $\lambda\in S:=\{1,i\}$, *where* $x_{3}= \lambda z$ ($z \in \mathcal {A}$) *and* (3.13). *If* $\delta(p e)=0$ *for all irrational numbers* *p*, *then* *δ* *is a linear left Jordan derivation*. *In this case* $\mathcal {A}$ *is a semiprime unital Banach algebra*, *δ* *is a linear derivation which maps* $\mathcal {A}$ *into the intersection of its center* $Z(\mathcal {A})$ *and its radical* $\operatorname{rad}(\mathcal {A})$.

### Proof

We first consider $\lambda=1$ in (3.16). We see by the result in [14] that there is a unique additive mapping $\mathcal{D}: \mathcal {A}\to \mathcal {A}$ defined by (3.15). In addition, $s\mathcal{D}(\frac{x}{s})=\mathcal{D}(x)$ for all $x \in \mathcal {A}$.

Also we set $\lambda=i$ in (3.16). And we take $x_{4}=x_{5}=\cdots=x_{l}=0$ in (3.16) and then let $x_{1}=x, x_{2}=y, s_{2}=t$ to have

3.17 $$\begin{aligned} \bigl\Vert i s\delta(x)+t\delta(y)+\delta(i z)\bigr\Vert \le \bigl\Vert \delta(i sx+ty+i z)\bigr\Vert +\varepsilon \end{aligned}$$

for all $x,y,z \in \mathcal {A}$. Putting $x=\frac{x}{s}, y=0$ and $z=-x$ in (3.17), we obtain

$$\begin{aligned} \biggl\Vert i s\delta \biggl(\frac{x}{s} \biggr)-\delta(i x)\biggr\Vert \le \varepsilon \end{aligned}$$

for all $x\in \mathcal {A}$, which shows that

$$\begin{aligned} \lim_{n \to\infty}\frac{1}{s^{n}}\biggl\Vert i s \delta \biggl( \frac {s^{n}x}{s} \biggr)-\delta\bigl(i s^{n}x\bigr)\biggr\Vert \le \lim_{n \to\infty} \frac {\varepsilon}{s^{n}}=0. \end{aligned}$$

Hence $i s\mathcal{D}(\frac{x}{s})=\mathcal{D}(i x)$. So we have $\mathcal{D}(i x)=i \mathcal{D}(x)$ for all $x\in \mathcal {A}$.

We have by (3.13)

$$\begin{aligned} \lim_{n \to\infty}\biggl\Vert \frac{\delta(s^{2n}(xy+yx))}{s^{2n}}- 2x \frac {\delta(s^{n}y)}{s^{n}}-2 y \frac{\delta(s^{n}x)}{s^{n}}\biggr\Vert \le \lim _{n \to\infty}\frac{\theta}{s^{2n}} =0. \end{aligned}$$

This implies that

3.18 $$\begin{aligned} \mathcal{D}(xy+yx)=2x\mathcal{D}(y)+2y\mathcal{D}(x) \quad\mbox{for all } x,y \in \mathcal {A}. \end{aligned}$$

Again, by virtue of (3.13), we see that

$$\begin{aligned} \lim_{n \to\infty}\biggl\Vert \frac{\delta(s^{n}(xy+yx))}{s^{n}}- 2x \delta (y)-2 y \frac{\delta(s^{n}x)}{s^{n}}\biggr\Vert \le\lim_{n \to\infty }\frac {\theta}{s^{n}} =0. \end{aligned}$$

This implies that

3.19 $$\begin{aligned} \mathcal{D}(xy+yx)=2x\delta(y)+2y\mathcal{D}(x) \quad\mbox{for all } x,y \in \mathcal {A}. \end{aligned}$$

Comparing (3.18) and (3.19), we arrive at $x\delta (y)=x\mathcal{D}(y)$ for all $x,y \in \mathcal {A}$. Since $\mathcal {A}$ contains the unit element, we find that $\mathcal {D}=\delta$. Equation (3.19) can be written

3.20 $$\begin{aligned} \delta(xy+yx)=2x\delta(y)+2y\delta(x) \quad\mbox{for all } x,y \in \mathcal {A}. \end{aligned}$$

Letting $x=y=e$ in (3.20), we have $\delta(e)=0$. Now we obtain by additivity of *δ* $\delta(q x)=q \delta(x)$ for all $q \in\mathbb{Q}$ and all $x \in \mathcal {A}$. So $\delta(q e)=q \delta (e)=0$ for all $q \in\mathbb{Q}$. This fact and the assumption of *δ* imply that $\delta(t e)=0$ for all $t \in\mathbb{R}$. Considering $y=t e$ in (3.20), we have $\delta(t x)=t \delta (x)$ for all $t \in\mathbb{R}$ and all $x \in \mathcal {A}$. Thus *δ* is $\mathbb{R}$-linear. Hence we see that

$$\begin{aligned} \delta(\mu x)=\delta\bigl((t_{1}+t_{2} i ) x\bigr)= \delta(t_{1} x)+t_{2} \delta( i x)=t_{1} \delta( x)+ t_{2} i\delta( x)=(t_{1} + t_{2} i)\delta( x)=\mu \delta( x) \end{aligned}$$

for all $\mu\in\mathbb{C}$ and all $x \in \mathcal {A}$. So we see that *δ* is $\mathbb{C}$-linear. In view of (3.20), we get

$$\delta\bigl(x^{2}\bigr)=2x\delta(x) \quad\mbox{for all } x \in \mathcal {A}. $$

Thereby *δ* is a linear left Jordan derivation.

On the other hand, if $\mathcal {A}$ is semiprime unital Banach algebra, then the rest of the proof is similar to the corresponding part of Theorem 3.1. □

### Theorem 3.6

*Let* $\mathcal {A}$ *be a semisimple Banach algebra*. *Assume that* $l \ge3$ *is a fixed integer and* $s_{1},s_{2}, \ldots, s_{l}$ *are fixed positive real numbers*, *where* $s_{1}=\lambda s$ ($s>1$), $s_{2}> 1$ *and* $s_{3}=1$. *Suppose that*, *for each* $k=0,1$, $\delta_{k} : \mathcal {A}\to \mathcal {A}$ *is a mapping with* $\delta_{k}(0)=0$ *such that*, *for some* $\varepsilon\ge0$,

3.21 $$\begin{aligned} \Biggl\Vert \sum_{j=1}^{l}s_{j} \delta_{k}(x_{j})\Biggr\Vert \leq\Biggl\Vert \delta _{k} \Biggl(\sum_{j=1}^{l}s_{j}x_{j} \Biggr)\Biggr\Vert +\varepsilon \end{aligned}$$

*for all* $x_{1}, x_{2}, \ldots, x_{l} \in \mathcal {A}$ *and all* $\lambda\in\mathbb{T}_{\varepsilon}$, *where* $x_{3}= \lambda z$ ($z \in \mathcal {A}$) *and*

3.22 $$\begin{aligned} &\bigl\Vert \delta_{0}(xy+yx)-2x\delta_{0}(y)-2y \delta_{0}(x)\bigr\Vert \leq\theta_{0}, \end{aligned}$$

3.23 $$\begin{aligned} &\bigl\Vert \delta_{1}(xy+yx)-x\delta_{1}(y)-y \delta_{1}(x)-x\delta _{0}(y)-y\delta_{0}(x)\bigr\Vert \leq\theta_{1} , \end{aligned}$$

*for some* $\theta_{0}, \theta_{1} \ge0$ *and all* $x,y \in \mathcal {A}$. *Then* $\delta_{1}$ *is a linear generalized left Jordan derivation associated with a linear left Jordan derivation* $\delta_{0}$. *In this case*, $\delta_{1}$ *is continuous*.

### Proof

It is well known that semisimple algebras are semiprime [18]. As we saw in the proof of Theorem 3.4, $\delta_{0}$ is a linear left Jordan derivation. In addition, we see that there exists a unique linear mapping $\mathcal {D}_{1}: \mathcal {A}\to \mathcal {A}$ defined by

3.24 $$\begin{aligned} \mathcal{D}_{1}(x):=\lim_{n\to\infty} \frac{\delta_{1}(s^{n}x)}{s^{n}} \quad \mbox{for all } x \in \mathcal {A}. \end{aligned}$$

According to (3.23) and (3.24), we see that

$$\begin{aligned} \lim_{n \to\infty}\biggl\Vert \frac{\delta_{1}(s^{n}(xy+yx))}{s^{n}}- x \delta_{1}(y)- y \frac{\delta_{1}(s^{n}x)}{s^{n}}-x\delta _{0}(y)-y\delta _{0}(x)\biggr\Vert \le\lim_{n \to\infty}\frac{\theta_{1}}{s^{n}} =0, \end{aligned}$$

which implies that

3.25 $$\begin{aligned} \mathcal{D}_{1}(xy+yx)=x\delta_{1}(y)+y \mathcal{D}_{1}(x)+x\delta _{0}(y)+y\delta_{0}(x) \quad\mbox{for all } x,y \in \mathcal {A}. \end{aligned}$$

So we obtain from (3.25)

3.26 $$\begin{aligned} \mathcal{D}_{1}(xy+yx)-x\delta_{0}(y)-y \delta_{0}(x)=x\delta _{1}(y)+y\mathcal{D}_{1}(x) \quad\mbox{for all } x,y \in \mathcal {A}. \end{aligned}$$

In particular, the left-side of equation (3.26) is a bilinear mapping. Lemma 3.3 guarantees that $\delta_{1}$ is linear. By (3.24), we have $\mathcal {D}_{1}=\delta_{1}$. Equation (3.25) gives

$$\delta_{1}\bigl(x^{2}\bigr)=x\delta_{1}(x)+x \delta_{0}(x) \quad\mbox{for all } x \in \mathcal {A}. $$

Thus $\delta_{1}$ is a linear generalized left Jordan derivation.

Therefore, since $\mathcal {A}$ is semisimple, we conclude that $\delta_{1}$ is continuous; see [19]. This completes the proof. □
